# Supplementary material for: “Because at school, you can become somebody” – The perceived health and economic returns on secondary schooling in rural Burkina Faso
Source: PLoS One. 2019 Dec 27;14(12):e0226911. doi: 10.1371/journal.pone.0226911 (PMC6934330; doi:10.1371/journal.pone.0226911)
Supplement: S1 File — (DOCX) [file pone.0226911.s001.docx]

**S1 File. Online Supplementary Materials**

In this supplementary appendix, we provide additional details related to our study, including:

- Text S1. Education and health context in Burkina Faso
- Text S2. Heterogeneity by school enrollment status and generations
- Figure S1. Map of study site and schools in Burkina Faso
- Table S1. Study inclusion and exclusion criteria
- Table S2. Selected characteristics of study schools

**Text S1. Education and Health Context in Burkina Faso**

Burkina Faso, a landlocked country in western sub-Saharan Africa, had a population of 17 million people in 2013 (WHO 2018), with over 70% living in rural area (Government of Burkina Faso). The total fertility rate is high (5.6 in 2013) and life expectancy at birth was around 60 years in 2016 (WHO 2018, World Bank 2018). Burkina Faso is one of the poorest countries in the world with around 40% of the population living in poverty (2009). Its GNI is 610 US dollars 2016 and the country ranks 181th out of 187 countries on the Human Development Index (World Bank 2018, Government of Burkina Faso 2018). With almost half its population aged under 15 (2013), Burkina Faso’s youth bears high potential for improving health and accelerating the country’s development (WHO 2018).

Although efforts to improve primary school completion have been to some extent successful (primary school enrolment rose from 12% in 1971 to 91% in 2016 (World Bank 2018)), secondary school completion remains very low (UNESCO 2017). Literacy is less than 30%. Access to secondary school varies highly according to regions. Junior secondary completion was 43% in Centre region compared to 4.5% in the Sahel region in the Northeast, suggesting substantial rural deprivation in education (Government of Burkina Faso 2018).

The school system of Burkina Faso, similar to the French school system, consists of six years of primary education, four years of lower secondary education (Junior Secondary School, JS) and three years of upper secondary education (Senior Secondary School, SS), in addition to three years of pre-primary education. Secondary school is divided into two ‘tracks’: i) general education and ii) technical/vocational education. Education is compulsory in Burkina Faso since 2007, from ages 6 to 16. In principle, school fees in public schools have been abolished, although schools are still in need to charge fees as state funding seems insufficient. The private sector plays a substantial role, covering between 36% and 63% of JS. School enrollment is lower in Burkina Faso than in comparable countries (Government of Burkina Faso 2018).

Principal causes of death remain mainly infectious diseases, including lower respiratory infections (14.3%), malaria (10.3%), and diarrheal diseases (6.2%) (WHO 2015). Knowledge of HIV prevention methods among young people aged 15-24 lies at 32% (2010 DHS).

**Text S2. Heterogeneity by School Enrollment Status and Generations**

Our study design allowed us to explore differences in perceptions across a number of participant categories. However, saturation might not be reached in each of these categories given smaller sample sizes and we therefore interpret these results with caution. Students seemed to show more awareness of schooling benefits vis-à-vis dropouts. Dropouts also often required more probing during the interviews to obtain detailed responses and avoid a closed question style. This was particularly the case when eliciting indirect and long-term benefits of schooling. In addition to being less aware of schooling benefits, school dropouts may have been intimated by interviewers who (by definition) were educated, and may have negatively judged adolescents who dropped out. In comparison, current students seemed to have more concrete ideas of the benefits of schooling as they expressed those better and were able to give concrete examples:

*“But why does school protect from that [risky behavior] in the long run?” (Interviewer)*

*“For example, when a dirty object [...] cuts you, that can cause tetanus... If you know that, you can beware of those objects; and then there are the things that cause cancer, you can beware of that, too; dirty waters; that’s why.” (17 years, male, student)*

Among the parental generation there was little difference between the perspectives of parents of dropouts vis-à-vis parents of current students. One reason could be that the parental generation had almost universally no formal schooling (6 out of 10 parents had no schooling whatsoever), regardless of the enrollment status of their children (**Table 1**). Parents of dropouts generally did not see fewer advantages to schooling than the parents of current students.

Figure S1. Map of study site and schools in Burkina Faso


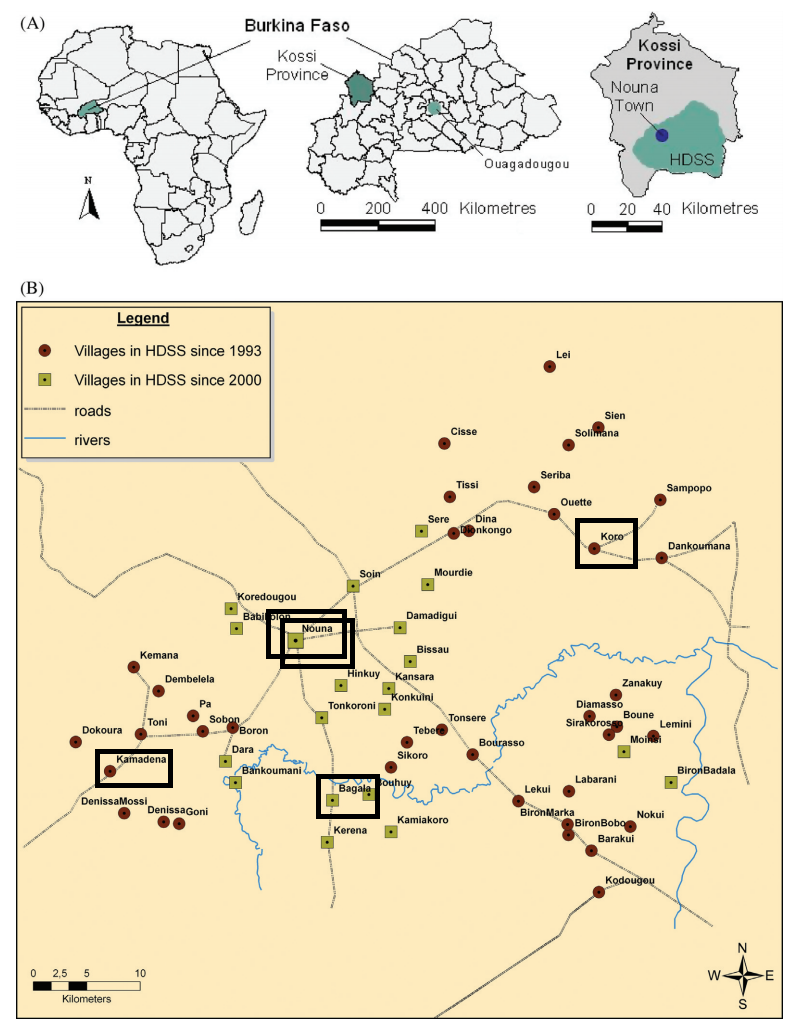


*Notes:* Geographical location of the Nouna Health and Demographic Surveillance System (HDSS) in Burkina Faso (A) and schools participating in the study (each frame represents a site with one participating school) (B). Source: Sie et al., 2010.

Table S1. Study inclusion and exclusion criteria

| **Inclusion criteria** | **Exclusion criteria** |
| --- | --- |
| Ages 13 or higher  Able to give informed consent (ages 18+) or ability to obtain informed consent from a legal guardian or parent (ages 13-17).  Individuals who are (i) attending formal school, (ii) drop-outs, (iii) parents of school-going age children, or (iv) those likely to be well-informed of school programming and context (e.g., school administrators). | Age under 13  Unable to give informed consent or obtain informed consent from legal guardian/parent. |

Table S2. Selected characteristics of study schools

*Notes:*  Most secondary schools in the HDSS are at the junior secondary school (JSS) level (19 out of a total of 28 secondary schools). Classes per teacher and mean of students per class were provided by school personnel. Abbreviations: JSS: junior secondary school.

**References for Supplementary Materials**

Burkina Faso. Ministères en charge de l'éducation et de la formation. Programme sectoriel de l'éducation et de la formation (PSEF) 2012-2021. [February 12, 2018]; Available from: <http://planipolis.iiep.unesco.org/fr/2013/programme-sectoriel-de-léducation-et-de-la-formation-psef-2012-2021-5626>.

Sié A, Louis VR, Gbangou A, Müller O, Niamba L, Stieglbauer G, et al. The Health and Demographic Surveillance System (HDSS) in Nouna, Burkina Faso, 1993-2007. *Global health action*. 2010;3(1):10. doi: 10.3402/gha.v3i0.5284.

World Bank. Data: Burkina Faso. [October 21, 2018]; Available from: <https://data.worldbank.org/country/burkina-faso>.

World Health Organization (WHO) Burkina Faso: WHO statistical profile. [October 21, 2018]; Available from: <http://www.who.int/gho/countries/bfa.pdf?ua=1>.

World Health Organization (WHO). Countries: Burkina Faso. Map. [December 26, 2018]; Available from: <http://www.who.int/countries/bfa/en/>.

UNESCO. Global education monitoring report, 2017/8: Accountability in education: Meeting our commitments. 2nd ed. Paris, France; 2017.
