# Supplementary material for: “Because at school, you can become somebody” – The perceived health and economic returns on secondary schooling in rural Burkina Faso
Source: PLoS One. 2019 Dec 27;14(12):e0226911. doi: 10.1371/journal.pone.0226911 (PMC6934330; doi:10.1371/journal.pone.0226911)
Supplement: Attachment — Submitted filename: Response to reviewers_18 Sept 2019.docx [file pone.0226911.s002.docx]

**Reviewers' comments:

Reviewer #1:**

*This paper provides an analysis of perceived benefits of secondary schooling in Burkina Faso. Among the strengths of this paper are its focus on secondary schooling; its inclusion of data from children who are in and also out of school, parents and diverse key informants; and its use of narratives to bring forward the voices and perspectives of local people. The paper also notes appropriately its limits regarding the small number of participants (e.g., only 7 school dropouts) and possible sampling biases.*

We thank the reviewer for these comments.

*The paper would be improved by more systematic attention to and analysis of gender dimensions. It would help to bring the findings related to gender out of the supplementary material and into the main body of the paper.*

We agree that the gender dimensions are an important component and have now discussed these in more detail in the Results and Discussion section of the paper. As recommended by the reviewer, we have moved findings related to gender out of the supplementary material and into the main text.

“We examine heterogeneity in perceptions by gender; as well as by school enrollment status and generations (**S2 Text**).” (p.10, revised manuscript)

“Heterogeneity in Perceptions by Gender

When comparing across genders, both female and male adolescents aspired equally to schooling, enabling them to gain employment. Nevertheless, the type of desired employment differed (e.g., females preferred to become a nurse vs. policeman). Female adolescents more often brought up barriers to secondary schooling compared to their male counterparts, such as the lack of (unisex) toilets, and security concerns. Similarly, marriage and pregnancy were also topics which came up exclusively among female adolescents and were reasons for dropping out of school. Females reported going to school while pregnant and/or with their children. All female dropouts, however, aspired going back to school, suggesting high perceived returns of secondary school attendance, despite these several additional concerns.” (p. 17, revised manuscript)

*Similarly, in developing recommendations, it would be useful to consider the likely need to develop different kinds of messages for women and men, respectively.*

We agree with the reviewer and have now further considered this in the Discussion section of the paper. We have also moved relevant material from the supplementary materials to the main text.

“Future (quasi-)experimental studies must assess whether efforts to raise awareness of the health and economic benefits of schooling could be a feasible approach to increasing school enrollment and attendance [45, 46]. The perceived benefits of schooling also differed by gender and generations, further suggesting the need for tailored interventions.” (p. 19, revised manuscript)

*Also, it would help to have a bit more analysis of the possible role of perceived benefits (and narratives surrounding them) in the causal nexus affecting decisions to go to school, stay in school, send children to school, etc. At present, the statements that perceived benefits may be causal or non-causal seem too binary and out of touch with what is likely a multi-causal system that extends over time and in which perceived benefits could have an influence at different points (e.g., decisions to send children to school, decisions to stay in school, decisions to work harder, etc.) and for different stakeholders (e.g., siblings, extended families, school age children, etc.) in a system of social decision making. It could also be useful to note further how, apart from the possible influence of perceived benefits, the perceived benefits may be inaccurate and reflect optimistic biases, among others.*

We thank the reviewer for these excellent suggestions. We have now further clarified these points in the Methods and Discussions sections of the paper, where we write:

“A key question is to what extent the perceptions of the benefits of schooling influence schooling investment decisions. In our analysis, students were generally more aware of the array of schooling benefits compared to school dropouts (**S2 Text**), suggesting that students might perceive higher returns than dropouts, leading them to invest more in schooling. However, with the current study design, we cannot ascertain whether this is a reason for them to stay at school or rather a consequence of schooling (or both). The perceived benefits of schooling are likely dynamic and the result of multiple factors. Schooling decisions are also made in the context of a complex social environment [44]. Future (quasi-)experimental studies must assess whether efforts to raise awareness of the health and economic benefits of schooling could be a feasible approach to increasing school enrollment and attendance [45, 46]. The perceived benefits of schooling also differed by gender and generations, further suggesting the need for tailored interventions.” (p. 19, revised manuscript)

“It is not clear to what extent the perceived benefits of schooling are accurate. The perceived returns, for instance, may reflect “optimistic bias”, because of unrealistic optimism about future life events [43]. Future research could compare the perceived returns with the measured returns on schooling, using longitudinal data routinely collected by the HDSS.” (p. 19, revised manuscript)

“The perceived benefits may impact schooling during different periods across the life course (e.g., as adolescent or parent), and for different stakeholders (e.g., siblings, extended families).” (Figure 1, revised manuscript)

References:

Sanfey A.G. (2007). Social decision-making: insights from game theory and neuroscience. *Science*. 26;318(5850):598-602

Weinstein, N. D. (1980). Unrealistic optimism about future life events. *Journal of Personality and Social Psychology*, 39(5), 806-820.

*Minor language improvements include: p. 19, line 9: ‘intimated’ should be ‘intimidated’*

We thank the reviewer for bringing this to our attention and have now corrected this sentence:

“Similarly, respondents, in particular adolescents who dropped out of school, may have felt intimidated by interviewers who were (by definition) relatively highly educated.” (p.20, revised manuscript)

*Abstract and after: ‘returns to schooling’ should be ‘returns on schooling’ since the former could refer to going back to school rather than the benefits of school participation.*

We have now edited this throughout the entire paper – e.g., see the following paragraphs:

“Because at school, you can become somebody” – The perceived health and economic returns on secondary schooling in rural Burkina Faso” (p.1, revised manuscript)

“In this study, we examine the perceived returns on secondary schooling among nearly 50 informants (school-age adolescents, parents, teachers, and key informants) in rural Burkina Faso, where secondary school completion is among the lowest in the world (<10%) [22].” (p.4, revised manuscript)

“The perceived returns on schooling are critical in schooling decision making and human capital investment but are not well understood.” (p.20, revised manuscript)

*I hope these comments are useful in making revisions.*

We thank the reviewer for these comments.

**Reviewer #2:**

*The topic for the paper is timely and of interest to diverse research and practice areas.*

We thank the reviewer for these comments.

*I do however believe the authors might have included a more robust literature review including what reasons for school dropout in primary schooling are understood and how this may/not inform this research on secondary schooling.*

We thank the reviewer for this excellent suggestion. We have now provided additional discussion on reasons for the lack of access to formal schooling and school dropout, where we write:

“A key reason for not enrolling in school is that formal schooling is “not deemed necessary”, suggesting that the perceived returns on attending formal schooling may play an important role in schooling decision-making [29]. Other commonly reported reasons for school absenteeism include financial barriers and distance to school.” (p.5, revised manuscript).

Reference:

National Institute of Statistics and Demography (2010), Enquete Integrale sur les Conditions de Vie des Menages [Integrated Household Living Conditions Survey], Burkina Faso, 2010.

*I suggest more discussion around the sampling and particular why such a small number of parents included.*

We have now further elaborated on our sampling approach in the Methods section (Morse 2016):

“The sample size was guided by the work of Morse (2016) [32]: 50 was considered as a number sufficient to gain saturation as returns on schooling in Burkina Faso represent explicit, apparent information which does not focus on sensitive topics (e.g., adolescent pregnancy). Furthermore, the sample of parents was chosen to be smaller due to higher expected quality of interviews with adults compared to those with adolescents.” (p.8, revised manuscript)

Reference:

Morse J.M. (2016). Determining Sample Size. *Qualitative Health Research*;10(1):3-5

*I suggest clarifying frequency of responses in the results section to get a better idea of how many times certain answers were given or at least to see which answers were more frequent as and perhaps rank answers in frequency to see which responses were most given vs. 1 or 2 people.*

We abstained from including frequencies of responses for the following reason. Qualitative research is an iterative process – in contrast to quantitative research – which questions rather than measures. This leads to a different depth between the first and last interviews as one learns along the process. Using semi-structured interviews, not every participant was asked exactly the same questions, or was asked the questions with the same intensity or same probing. This would lead to a distortion of frequency results. We would be happy, however, to make further changes at the behest of the editor.

Reference:

Auerbach, C. F., Silverstein, L. B. (2003). Qualitative data: An introduction to coding and analysis. New York, NY, US: New York University Press.

*The authors may benefit from expanding their discussion of grounded theory, why they chose this line of inquiry and whether such an approach should be replicated in future studies. What other methods might be used in future studies and why? Given that this hard-to-reach population is understudied please the authors may elaborate on what future studies might work best.*

We thank the reviewer for these excellent suggestions. We have now provided more discussion on our analytical approach. Additionally, we have now moved the theoretical framework (**Figure 1**), which underpins our study, out of the supplementary material and inserted it into the main text to further clarify our approach and findings. As recommended by the reviewer, we have also further elaborated on the implications of our findings for research, including possible future study designs.

“Our analysis proceeded in two steps. The first step was content analysis informed by grounded theory [32, 35, 36]. We did not impose a framework but developed one in an inductive approach building on our coding results (**Figure 1**) [36]. Interview passages were coded to mutually exclusive topics (codes) arising from the interview data, using a codebook with descriptions for each topic to facilitate consistent use of codes by study team members throughout the study period. Major topics that arose from the data included health knowledge acquired at school (such as about general hygiene); interactions with the formal health system; language skills; employment; success and recognition; quality of life; as well as increased ability to provide support to family members and, in particular, parents in their old-age. The second step in our analysis was to group coded items by major categories based on commonalities and patterns. Major categories included health, economic factors, and key abilities and skills [37].” (p.8, revised manuscript)

“It is not clear to what extent the perceived benefits of schooling are accurate. The perceived returns, for instance, may reflect “optimistic bias”, because of unrealistic optimism about future life events [43]. Future research could compare the perceived returns with the measured returns on schooling, using longitudinal data routinely collected by the HDSS.” (p.19, revised manuscript)

“A key question is to what extent the perceptions of the benefits of schooling influence schooling investment decisions. In our analysis, students were generally more aware of the array of schooling benefits compared to school dropouts (**S2 Text**), suggesting that students might perceive higher returns than dropouts, leading them to invest more in schooling. However, with the current study design, we cannot ascertain whether this is a reason for them to stay at school or rather a consequence of schooling (or both). The perceived benefits of schooling are likely dynamic and the result of multiple factors. Schooling decisions are also made in the context of a complex social environment [44]. Future (quasi-)experimental studies must assess whether efforts to raise awareness of the health and economic benefits of schooling could be a feasible approach to increasing school enrollment and attendance [45, 46]. The perceived benefits of schooling also differed by gender and generations, further suggesting the need for tailored interventions.” (p.19, revised manuscript)

“Figure 1. Conceptual framework underpinning the study.” (p.9, revised manuscript)

*Notes:* Author’s conceptual framework where the perceived benefits of secondary school may impact school attendance and in turn the measured education gains (e.g., increased educational attainment), health gains (e.g., improved sexual and reproductive health outcomes), and household financial gains (e.g., increased employment opportunities, higher salary, financial independence). The perceived benefits may impact schooling during different periods across the life course (e.g., as adolescent or parent), and for different stakeholders (e.g., siblings, extended families).

References:

Charmaz K. (2006). Constructing grounded theory. London; Thousand Oaks, Calif.: Sage Publications. xiii, 208 p.p.

Snilstveit B, Stevenson J, Phillips D, Vojtkova M, Gallagher E, Schmidt T, et al. (2015). Interventions for improving learning outcomes and access to education in low- and middleincome countries: a systematic review, 3ie Systematic Review 24. London.

*The limitations section as it provides a well thought out commentary of its limits. In addition, the policy and practice implications are well considered.*

We thank the reviewer for these comments.
