# Supplementary material for: “Because at school, you can become somebody” – The perceived health and economic returns on secondary schooling in rural Burkina Faso
Source: PLoS One. 2019 Dec 27;14(12):e0226911. doi: 10.1371/journal.pone.0226911 (PMC6934330; doi:10.1371/journal.pone.0226911)
Supplement: Attachment — Submitted filename: Response to Reviewers 6 Nov 2019.docx [file pone.0226911.s003.docx]

**Reviewer #2:**

*Question on Table 1. Characteristics of study respondents, under Age of School-age adolescent Dropouts, there seems to be one subject not accounted for? the total of dropouts I believe was 9 however under ages I only count 8? There are 6 under ages 15-17, 1 ages 18-20 and 1 Missing however all other totals in that column equal 9*

We thank the reviewer for bringing this to our attention. The age of one dropout ages 18-20 was not accounted for and we have now corrected this in Table 1 (so that the total in its column equals 9):

“Table 1. Selected characteristics of study respondents (*N* = 49)”

| ***Characteristics*** |  | **School-age adolescents (n=19)** | | **Parents (n=10)** | | **Teachers (n=10)** | **Other (n=10)** |  | **Total n (%)** |
| --- | --- | --- | --- | --- | --- | --- | --- | --- | --- |
|  |  |  |  |  |  |  |  |  |  |
|  |  |  |  |  |  |  |  |  |  |
|  |  | *Dropout* | *Enrolled* | *Dropout* | *Enrolled* |  |  |  |  |
| Age |  |  |  |  |  |  |  |  |  |
| <15 |  | 0 | 2 | 0 | 0 | 0 | 0 |  | 2 (4) |
| 15 – 17 |  | 6 | 4 | 0 | 0 | 0 | 0 |  | 10 (20) |
| 18 – 20 |  | 2 | 0 | 0 | 0 | 0 | 1 |  | 3 (6) |
| 21 – 40 |  | 0 | 2 | 1 | 0 | 9 | 3 |  | 15 (31) |
| >40 |  | 0 | 0 | 3 | 3 | 0 | 5 |  | 11 (22) |
| Missing |  | 1 | 2 | 1 | 2 | 1 | 1 |  | 8 (16) |
| Gender |  |  |  |  |  |  |  |  |  |
| Female |  | 4 | 5 | 0 | 0 | 3 | 1 |  | 13 (27) |
| Male |  | 5 | 5 | 5 | 5 | 7 | 9 |  | 36 (73) |
| Missing |  | 0 | 0 | 0 | 0 | 0 | 0 |  | 0 (0) |
| Religion |  |  |  |  |  |  |  |  |  |
| Muslim |  | 6 | 4 | 3 | 4 | 4 | 7 |  | 28 (57) |
| Christian |  | 3 | 6 | 2 | 0 | 5 | 3 |  | 19 (39) |
| Animist |  | 0 | 0 | 0 | 1 | 1 | 0 |  | 2 (4) |
| Missing |  | 0 | 0 | 0 | 0 | 0 | 0 |  | 0 (0) |
| School level (years) |  |  |  |  |  |  |  |  |  |
| 0 |  | 0 | 0 | 2 | 4 | 0 | 2 |  | 8 (16) |
| 1 – 6 |  | 0 | 0 | 2 | 0 | 0 | 1 |  | 3 (6) |
| 7 – 10 |  | 9 | 10 | 0 | 1 | 0 | 0 |  | 20 (41) |
| 11 – 13 |  | 0 | 0 | 0 | 0 | 0 | 1 |  | 1 (2) |
| >13 |  | 0 | 0 | 0 | 0 | 10 | 6 |  | 16 (33) |
| Missing |  | 0 | 0 | 1 | 0 | 0 | 0 |  | 1 (2) |
| Mother tongue |  |  |  |  |  |  |  |  |  |
| Bwamu |  | 3 | 4 | 2 | 1 | 2 | 1 |  | 13 (27) |
| Dafing |  | 4 | 2 | 1 | 1 | 0 | 1 |  | 9 (18) |
| Mooré |  | 0 | 1 | 0 | 1 | 2 | 5 |  | 9 (18) |
| Dioula |  | 1 | 0 | 1 | 0 | 1 | 2 |  | 5 (10) |
| Other |  | 1 | 3 | 1 | 2 | 5 | 1 |  | 13 (27) |
| Missing |  | 0 | 0 | 0 | 0 | 0 | 0 |  | 0 (0) |
| French |  |  |  |  |  |  |  |  |  |
| Yes |  | 7 | 10 | 3 | 0 | 10 | 9 |  | 39 (80) |
| No |  | 2 | 0 | 2 | 5 | 0 | 1 |  | 10 (20) |
| Missing |  | 0 | 0 | 0 | 0 | 0 | 0 |  | 0 (0) |
|  |  |  |  |  |  |  |  |  |  |

*Notes:* Table shows selected characteristics of study respondents. Data are number of individuals. Category ‘Other’ includes key informants that were interviewed during the second stage – e.g., students, tutors, teachers, school directors, member of a students’ parent association, as well as health workers. Structural characteristics on schools included in the study are available in S2 Table.

*Was there any analysis in Table 2 about which answers were most common (hierarchy of responses?) as I believe categories were exclusive?*

We abstained from including frequencies of responses in Table 2 as qualitative research is an iterative process – in contrast to quantitative research – which questions rather than measures. We have now further clarified our analysis in Table 2 in the Methods section of the paper, where we write:

“Lastly, we abstained from including frequencies of responses (a hierarchy of responses) since qualitative research is an iterative process – in contrast to quantitative research. This leads to a different depth between the first and last interviews as one learns along the process. Using semi-structured interviews, not every participant was asked exactly the same questions, or was asked the questions with the same intensity or same probing. This would lead to a distortion of frequency results.” (p.9, revised manuscript)

References:

Auerbach, C. F., Silverstein, L. B. (2003). Qualitative data: An introduction to coding and analysis. New York, NY, US: New York University Press.

Saldaña, J. (2009). The coding manual for qualitative researchers. Thousand Oaks, CA, : Sage Publications Ltd.
